# CXCL4 contributes to host defense against acute *Pseudomonas aeruginosa* lung infection

**DOI:** 10.1371/journal.pone.0205521

**Published:** 2018-10-08

**Authors:** Lei Yue, Zheng Pang, Hua Li, Ting Yang, Lei Guo, Longding Liu, Junjie Mei, Xia Song, Tianhong Xie, Ye Zhang, Xin He, Tong-Jun Lin, Zhongping Xie

**Affiliations:** 1 The Institute of Medical Biology, Chinese Academy of Medical Sciences and Peking Union Medical College, Kunming, Yunnan, China; 2 Department of Pathology, Dalhousie University, Halifax, Nova Scotia, Canada; 3 Department of Microbiology and Immunology, Dalhousie University, Halifax, Nova Scotia, Canada; 4 Department of Pediatrics, Dalhousie University, Halifax, Nova Scotia, Canada; 5 Beatrice Hunter Cancer Research Institute, Halifax, Nova Scotia, Canada; Washington State University, UNITED STATES

## Abstract

Platelets have been implicated in pulmonary inflammation following exposure to bacterial stimuli. The mechanisms involved in the platelet-mediated host response to respiratory bacterial infection remain incompletely understood. In this study, we demonstrate that platelet-derived chemokine (C-X-C motif) ligand 4 (CXCL4) plays critical roles in a mouse model of acute bacterial pneumonia using *Pseudomonas aeruginosa*. Platelets are activated during *P*. *aeruginosa* infection, and mice depleted of platelets display markedly increased mortality and impaired bacterial clearance. CXCL4 deficiency impairs bacterial clearance and lung epithelial permeability, which correlate with decreased neutrophil recruitment to BALF. Interestingly, CXCL4 deficiency selectively regulates chemokine production, suggesting that CXCL4 has an impact on other chemokine expression. In addition, CXCL4 deficiency reduces platelet-neutrophil interactions in blood following *P*. *aeruginosa* infection. Further studies revealed that platelet-derived CXCL4 contributes to the *P*. *aeruginosa*-killing of neutrophils. Altogether, these findings demonstrate that CXCL4 is a vital chemokine that plays critical roles in bacterial clearance during *P*. *aeruginosa* infection through recruiting neutrophils to the lungs and intracellular bacterial killing.

## Introduction

*Pseudomonas aeruginosa* is an environmentally ubiquitous Gram-negative bacterial pathogen and one of the leading causes of morbidity and mortality among cystic fibrosis (CF) patients and immunocompromised individuals[1]. Effective bacterial clearance relies not only on neutrophil-mediated bacterial phagocytosis and killing[1], but also coordination of multiple immune effector cells, such as macrophages[2], NK cells[3], T cells[4] and B cells[5], which play critical roles in regulation of neutrophil recruitment to infection site by secreting specific chemokines during *P*. *aeruginosa* lung infection.

Platelets have been known to be essential for hemostasis and wound healing since their first identification[6]. An increasing number of studies on important roles in anti-microbial responses and inflammation have been reported in past decade[6–9]. As a first line of host defense, platelets act as primitive immune cells, interacting with invading bacterial pathogens or recruiting immune cells[10]. Previous studies have reported that *P*. *aeruginosa* phospholipase C[11], EF-Tu[12] and ExoU[13] cause platelet activation, implying that platelets may play an important role during *P*. *aeruginosa* infection. However, the mechanisms involved in platelet-mediated bacterial clearance during *P*. *aeruginosa* infection are not fully understood.

Chemokine (C-X-C motif) ligand 4 (CXCL4), also known as platelet factor 4 (PF4), was the first CXC chemokine discovered. CXCL4 is highly abundant in platelet granules, and it facilitates chemotaxis for neutrophils and monocytes through binding to the chemokine receptor CXCR3[14]. Experimental evidences have shown that CXCL4 plays important roles in angiogenesis[15], tissue repair[16], and regulation of tissue damage in complex inflammatory disease, such as systemic sclerosis[17], melanoma[18], and antimalarial models[19]. Recent evidences have shown that CXCL4 participates in inflammation, innate immunity[8], and particularly bacterial clearance[20, 21]. CXCL4 binds to bacteria[22, 23] and activates neutrophils through multiple intracellular signals and pathways[24]. Previous studies have shown essential roles for PI3K[25], Syk[26], JNK[27], p38[28] and JAK/STAT[29, 30] signaling during host defense against *P*. *aeruginosa*, suggesting a potential role of CXCL4 in *P*. *aeruginosa* lung infection.

In this study, we found that CXCL4 deficiency impairs bacterial clearance and lung epithelial permeability, which correlate with decreased neutrophil recruitment to BALF. CXCL4 deficiency selectively regulates neutrophil chemokine responses without affecting inflammatory cytokine production. More importantly, CXCL4 deficiency reduces platelet-neutrophil interactions in the blood following *P*. *aeruginosa* infection. Moreover, platelet-derived CXCL4 contributes to the *P*. *aeruginosa*-killing capability of neutrophils. Interestingly, CXCL4 deficiency has no effect on the mortality, body weight and the recruitment of other inflammatory cells to the lungs after *P*. *aeruginosa* infection. Altogether, these results demonstrate that CXCL4 plays critical roles in regulation of neutrophil recruitment to the lungs for *P*. *aeruginosa* clearance through facilitating platelet-neutrophil interactions and promoting intracellular killing.

## Materials and methods

### Animals

The CXCL4^-/-^ mice[31] and WT control C57BL/6 mice were originally provided by Dr. G. Scott Worthen at Children’s Hospital of Philadelphia. Animal care and experimental protocols were reviewed and approved by the Yunnan Provincial Experimental Animal Management Association and the Experimental Animal Ethics Committee of the Institute of Medical Biology, Chinese Academy of Medical Sciences, according to the national guidelines on animal work in China. Animals were housed in specific pathogen free facilities and anesthetized with ketamine to minimize suffering during relevant procedures.

### Antibodies

APC-conjugated rat anti-mouse CD41(IgG1, clone MWReg30), PE-conjugated rat anti-mouse CD62P (IgG1, clone Psel.KO2.3), PE-conjugated rat anti-mouse Ly6G (IgG2a, clone 1A8), APC-conjugated rat anti-mouse F4/80 (IgG2a, clone BM8), and an isotype control were purchased from eBioscience (San Diego, CA).

### Bacterial preparation

*P*. *aeruginosa* was cultured in Luria-Bertani broth at 37°C and harvested when the culture reached an OD value between 2.5 to 3 at 600 nm (early stationary phase). Bacteria were washed in phosphate buffer and resuspended in saline for *in vivo* experiments or PBS for *in vitro* assays. The *P*. *aeruginosa* strain 8821 (a gift from A. Chakrabarty, University of Illinois, Chicago, IL) used in cell culture assays was killed using an antibiotic mixture (50 U/ml penicillin, 50 U/ml streptomycin, 100 μg/ml piperacillin, 100 μg/ml ceftazidime, and 200 μg/ml gentamycin).

### Animal survival

To determine animal survival after infection, mice were intranasally infected with 1×10^9^ CFU of *P*. *aeruginosa* 8821. The mice were kept in the isolated room and monitored twice a day, and the disease scores were recorded for 7 days according to the following disease scoring system: (1) Physical appearance (0—normal; 1—lack of grooming; 2 –rough hair coat; 3—very rough hair coat); (2) Posture (0—normal; 1—sitting in hunched position; 2—hunched posture, head resting on floor; 3—lying prone on cage floor/unable to maintain upright posture); (3) Activity/Behavior (0—normal; 1—somewhat reduced/minor changes in behavior; 2—above plus change in respiratory rate or effort; 3—moves only when stimulated); (4) Appetite (0—normal; 1—reduced appetite; 2—not eating since last check point; 3 –not eating for last 2 check points); (5) Hydration (0—normal; 1—mildly dehydrated; 2—moderately dehydrated; 3—severely dehydrated); (6) Body weight (0 - <5% change from pre-infection weight); 1 - < 10% weight change; 2–10–15% weight change: 3–15–19% weight change); (7) Body temperature (ventral surface temp) (0–33–34°C; 1–28–32.5°C; 2 -25-27.5°C; 3 - < 24.5°C). Total possible score = 21. The animals were euthanized when the score reached or was greater than 15.

### Platelet depletion

For circulating platelet depletion experiments, sixteen hours before infection with *P*. *aeruginosa*, wild-type mice were given an intraperitoneal injection of 50 μL of rabbit anti-mouse platelet serum (Cedarlane, Burlington, NC), and normal rabbit serum was use for control mice. Whole blood cells were collected, and the depletion of the platelet was confirmed by flow cytometry (more details are described below).

### Lung infection with *P*. *aeruginosa* and collection of lungs, BALF and blood

*P*. *aeruginosa* strain 8821, a mucoid strain isolated from a cystic fibrosis patient, was used [32]. Mice were intranasally infected with 1×10^9^ CFU for 24 h. Blood (100 μL) was collected from a facial vein in an RNase-free 1.5 mL tube for serum isolation or a 1.5 mL tube with anticoagulant for flow cytometry. 10 mL PBS was infused into the heart to remove blood from the lungs. Bronchoalveolar lavage fluid (BALF) was obtained by lavaging the lungs with 1 mL of PBS containing soybean trypsin inhibitor (100 μg/mL), and then 2 x 1ml PBS was used to wash the alveoli. The lavaged lung tissue was obtained for histology study, cytokine detection, myeloperoxidase (MPO) assay, and counting of bacterial CFU.

Lung tissue was homogenized in 50 mM HEPES buffer (4 μL/mg lung) containing soybean trypsin inhibitor (100 μg/mL). For counting bacterial CFU, 10 μL homogenate was plated onto an agar dish and incubated for 24 h at 37°C. The lung homogenate was centrifuged at 4°C for 20 min at 18,000 × g. The supernatant was stored at -80°C for subsequent cytokine analysis. The pellet was resuspended and homogenized in 0.5% cetyltrimethylammonium chloride (4 μL/mg lung) and centrifuged, as described above. The cleared supernatant was used for the MPO assay.

BALF (10 μL) was plated onto an agar dish and incubated for 24 h to count CFU. For detection of cytokines and MPO activity, BALF was centrifuged at 1 200 × g for 5 min at 4°C. The supernatants were used for cytokine analysis. The pellets were resuspended in 1 mL NH_4_Cl (0.15 M) and centrifuged as before to lyse red blood cells. The supernatants were discarded, and the pellets were resuspended in 0.5% cetyltrimethylammonium chloride (250 μL/ mouse) and then centrifuged. The cleared extracts were used for the MPO assay. In addition, the pellets after erythrocyte lysis could also be used for flow cytometry.

Blood (10 μL) was plated onto an agar dish and incubated for 24 h for CFU determination. To isolate serum, blood in a clean tube was set at room temperature for 60 min and then centrifuged at 3500 rpm for 5 min. The serum was collected from the top of the tube. To isolate blood cells, Erythrocyte lysis buffer (BD Biosciences) was added to the blood that is in tubes containing anticoagulant. Blood cells were spun down at 300 g for 10 min and resuspended for flow cytometry.

### Cytokine production

The concentrations of IL-1β, TNF, IL-6, MIP2, KC and LIX in the BALF, lungs, and culture supernatants were determined by ELISA as described previously[33] using DuoSet Ab pairs from R&D Systems (Minneapolis, MN). Briefly (*e*.*g*. IL-6 ELISA), 96-well plates were coated with anti-mouse IL-6 for 16–20 h at 4°C. Nonspecific binding to the plates was blocked using a 1% bovine serum albumin solution in PBS for 1 h at room temperature. A total of 50 μL/well IL-6 standard and samples were added to the plate and incubated for 18–20 h at 4°C. Biotinylated anti-murine IL-6 were added to each well and incubated for 1 h at room temperature. 100 μL/well of Streptavidin-HRP was added for 30 minutes at room temperature according to the manufacturer’s instructions. 100 μL/well of 1X TMB Solution was added to each well, and it was stopped by 100 μL Stop Solution (0.5 M H_2_SO_4_). Read the plate at 450 nm and analyze data.

### MPO assay

The MPO assay was used to determine the infiltration of neutrophils into the lungs of the mice as described previously [34]. Briefly, samples in duplicate (75 μL) were mixed with equal volumes of the substrate (3,3’,5,5’—tetramethyl—benzidine dihydrochloride, 3 mM; Resorcinol, 120 μM; and H_2_O_2_, 2.2 mM) for 2 min. The reaction was stopped by adding 150 μL of 2M H_2_SO_4_. The OD was measured at 450 nm.

### *In vivo* permeability measurement

The mice were intranasally infected with *P*. *aeruginosa* strain 8821 (1×10^9^ CFU/mouse) for 24 h. Eighteen hours prior to sacrifice, the mice were given a 400 μL i.p. injection of 0.5% Evans blue dye (Sigma Aldrich) in a phosphate buffer solution. The blood, BALF and lungs were collected at 24 h post infection (hpi). The serum was diluted 1:20 in phosphate buffer solution. Dye leakage into the BALF was assessed and is presented as the BALF permeability index, which is the ratio of BALF to serum at OD620 (1:20 dilution). Evan blue dye was then extracted from lung tissue by homogenization. The lung homogenate was centrifuged at 4°C for 20 min at 18,000 × g, and the supernatant was collected. The dye leaked into the interstitial space was assessed and presented as the lung permeability index, which is the ratio of lung to serum at OD620 (1:20 dilution).

### Flow cytometry

BALF and blood were collected as described above. Single-cell suspensions were washed after erythrocytes were lysed (BD Biosciences). Cells were incubated with BD Fc block (BD Biosciences) for 10 min, and cell surface staining was performed with antibodies against CD41, CD62P, Ly6G, F4/80 and isotype control for 30 min. Flow cytometric data were collected on a BD Accuri C6 Flow Cytometer (BD Biosciences) and analyzed using BD Accuri C6 version 1.0 (BD Biosciences) and FlowJo version 10.1 (Ashland, OR) software.

### Measurement of NO production

Mouse bone marrow-derived neutrophils were isolated from mice following the protocol of a Mouse Neutrophil Negative Selection Kit (STEMCELL Technologies Inc.,). Platelets were isolated from whole blood samples according to reported method[35]. Neutrophil or platelet were left untreated (NT) or exposed to *P*. *aeruginosa* strain 8821 (MOI = 10) at 37°C. Cell-free supernatants were collected and analyzed for NO production following the protocol of Griess Reagent System Kit (Promega).

### Phagocytosis assay

The neutrophils were counted and then incubated with *P*. *aeruginosa* 8821 (MOI = 10), which was opsonized with 10% mouse serum, at 37°C for 30 min. The neutrophil pellet was washed with PBS and then treated with PBS containing 0.1% trypsin and 0.02% EDTA for 15 min at room temperature. Neutrophils were resuspended in PBS containing 10% mouse serum. Specimens were prepared using a Cytospin 4 Cytocentrifuge (Thermo Fisher Scientific, Waltham, MA). The cytospin specimens were then stained with a Diff-Quik stain set (Siemens Healthcare Diagnostics Inc., Newark, DE) and examined under oil immersion. The number of bacteria engulfed by 100 randomly selected neutrophils was counted. The phagocytic activity was measured according to the phagocytosis index. The phagocytosis index was calculated as the average number of bacteria/neutrophils counted.

### Intracellular bacterial killing assay

Neutrophils were isolated as described above and incubated with *P*. *aeruginosa* 8821 (opsonized with mouse serum) at 37°C for 1 h. Gentamycin was added at a final concentration of 200 mg/ml to kill extracellular bacteria for 3 h. Then, the neutrophils were washed with PBS and lysed with PBS containing 0.1% Triton X-100. The samples were serially diluted and spread onto Luria broth (LB) agar plates. Colony numbers were determined after overnight incubation at 37°C.

### Histology

Mouse lungs were fixed in 10% formalin overnight and then in 100% ethanol for paraffin embedding and sectioning. The sections were deparaffinized with CitriSolv (Thermo Fisher Scientific), rehydrated in decreasing concentrations of ethanol, and stained with Harris H&E to illustrate lung histology.

### Statistics

The data are presented as the means ± SEM of the indicated number of experiments. Statistical significance between multiple treatments was determined by one-way analysis of variance and Tukey’s post hoc honest significance test. Alternatively, when two independent variables were analyzed, two-way analysis of variance and Bonferroni’s multiple-comparison test were used. Statistical analysis was performed using GraphPad Prism software version 5.04 (GraphPad Software Inc., La Jolla, CA). Differences were considered significant at *p < 0.05, **p < 0.01, and ***p <0.001.

## Results

### 1. Platelet depletion impairs host defense against acute *P*. *aeruginosa* pulmonary infection in mice

**Fig 1:**
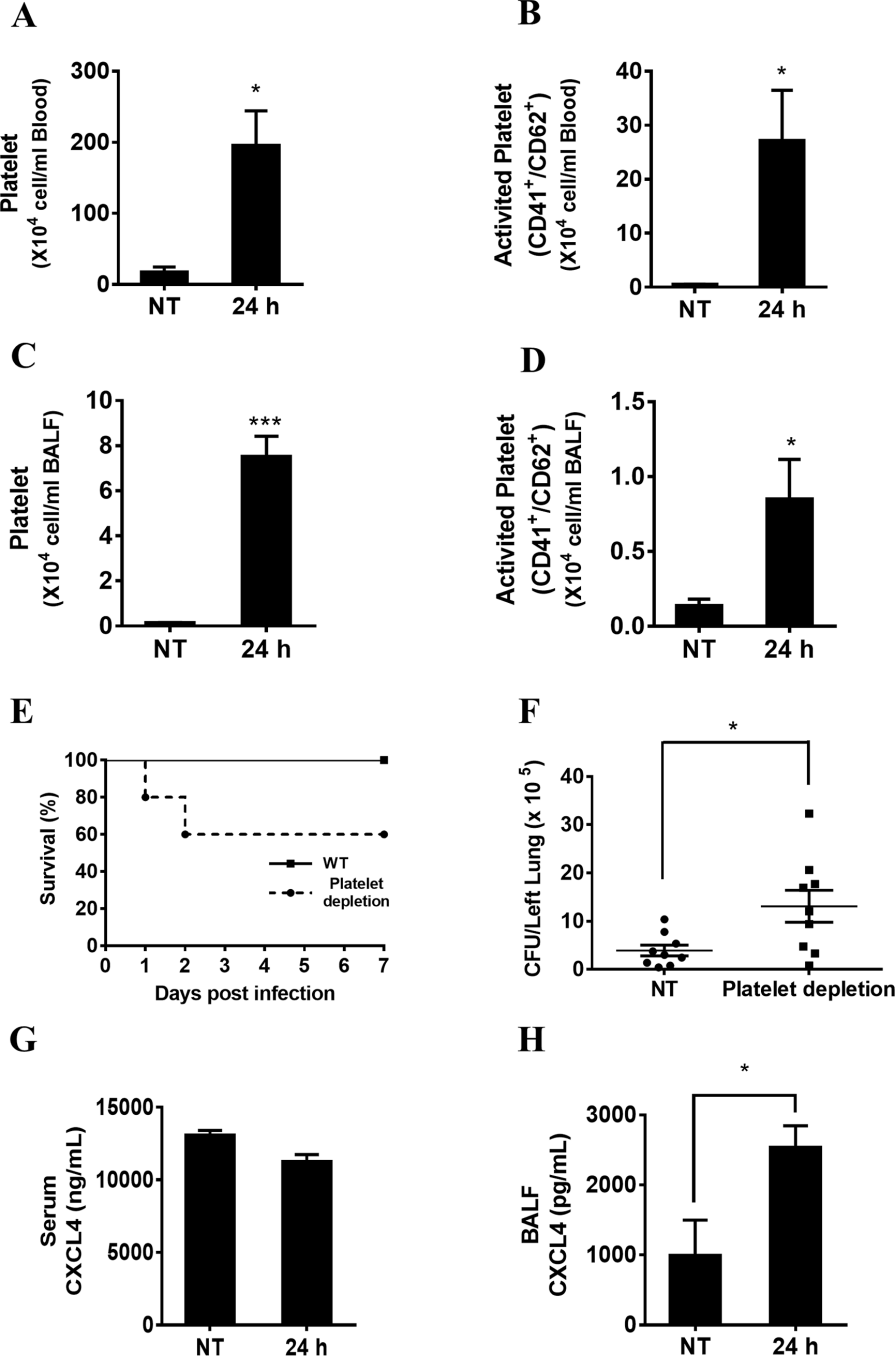
Platelet depletion impairs host defense against acute *P*. *aeruginosa* pulmonary infection in mice. (A, B, C, D) Platelets were activated after *P*. *aeruginosa* lung infection. Wild-type mice were intranasally infected with 1×10^9^ CFU of *P*. *aeruginosa* strain 8821 or an equivalent volume of saline (NT). Blood and BALF were collected 24 hpi. Platelet activation in the blood and BALF was assessed via flow cytometry (n = 4 ± SEM, *p < 0.05, ***p < 0.001). (E) Platelet depletion decreases animal survival following *P*. *aeruginosa* infection. Sixteen hours prior to infection with *P*. *aeruginosa*, wild-type mice were administered an intraperitoneal injection of 50 μL of rabbit anti-mouse platelet serum. Wild-type and platelet-depleted mice were intranasally infected with saline (NT) or 1×10^9^ CFU of *P*. *aeruginosa* strain 8821. Animal survival was monitored for 7 days post infection (n = 10 ± SEM). (F) Platelet depletion impairs bacterial clearance following *P*. *aeruginosa* lung infection. Wild-type and platelet-depleted mice were intranasally infected with 1×10^9^ CFU of *P*. *aeruginosa* strain 8821 for 24 h. Lungs were collected at 24 hpi. Serial dilutions of homogenized lung tissues were streaked onto LB agar plates and incubated for 24 h at 37°C. The resulting colonies were counted to determine the bacterial load (n = 9 ± SEM, *p < 0.05). (G, H) CXCL4 is mainly activated in the location that *P*. *aeruginosa* infected. Wild-type mice were intranasally infected with 1×10^9^ CFU of *P*. *aeruginosa* strain 8821 for 24 h. Serum and BALF supernatants were collected for determining CXCL4 production (n = 3 ± SEM, **p < 0.01, ***p < 0.001).

To study whether platelets can be activated during acute *P*. *aeruginosa* infection *in vivo*, wild-type mice were intranasally infected with *P*. *aeruginosa* strain 8821. The results showed that the number of platelets and activated platelets were both increased in the blood (Fig 1A and 1B) and BALF (Fig 1C and 1D) at 24 hpi compared to untreated samples using flow cytometry assays. To determine the biological role of platelets, anti-platelet serum was used to block platelet function (Figure A-B in S1 Fig). Platelet-depleted and wild-type mice were intranasally infected with *P*. *aeruginosa*. The bacterial burden was assessed in the lungs, BALF and blood at 24 hpi by CFU counting. Mice depleted of platelets displayed increased mortality (Fig 1E) and bacterial load in the lungs (Fig 1F) combined with severe lung damage based on histopathological assay (S2 Fig). No significant bacterial load change was detected in the BALF and blood (Figure A-B in S3 Fig) of platelet-depleted mice compared with wild-type mice. These data suggest that platelet depletion impairs bacterial clearance and increases mortality during acute *P*. *aeruginosa* pulmonary infection *in vivo*. CXCL4 is secreted from internal granules upon platelet activation[36]. We further measured the level of CXCL4 secretion in serum and BALF from wild-type mice after *P*. *aeruginosa* infection. No detectable changes of CXCL4 levels were observed in wild-type serum at 24 hpi (Fig 1G). However, the CXCL4 levels in the BALF (Fig 1H) were significantly elevated at 24 hpi.

### 2. CXCL4 deficiency impairs bacterial clearance and airway epithelial permeability after *P*. *aeruginosa* lung infection

**Fig 2:**
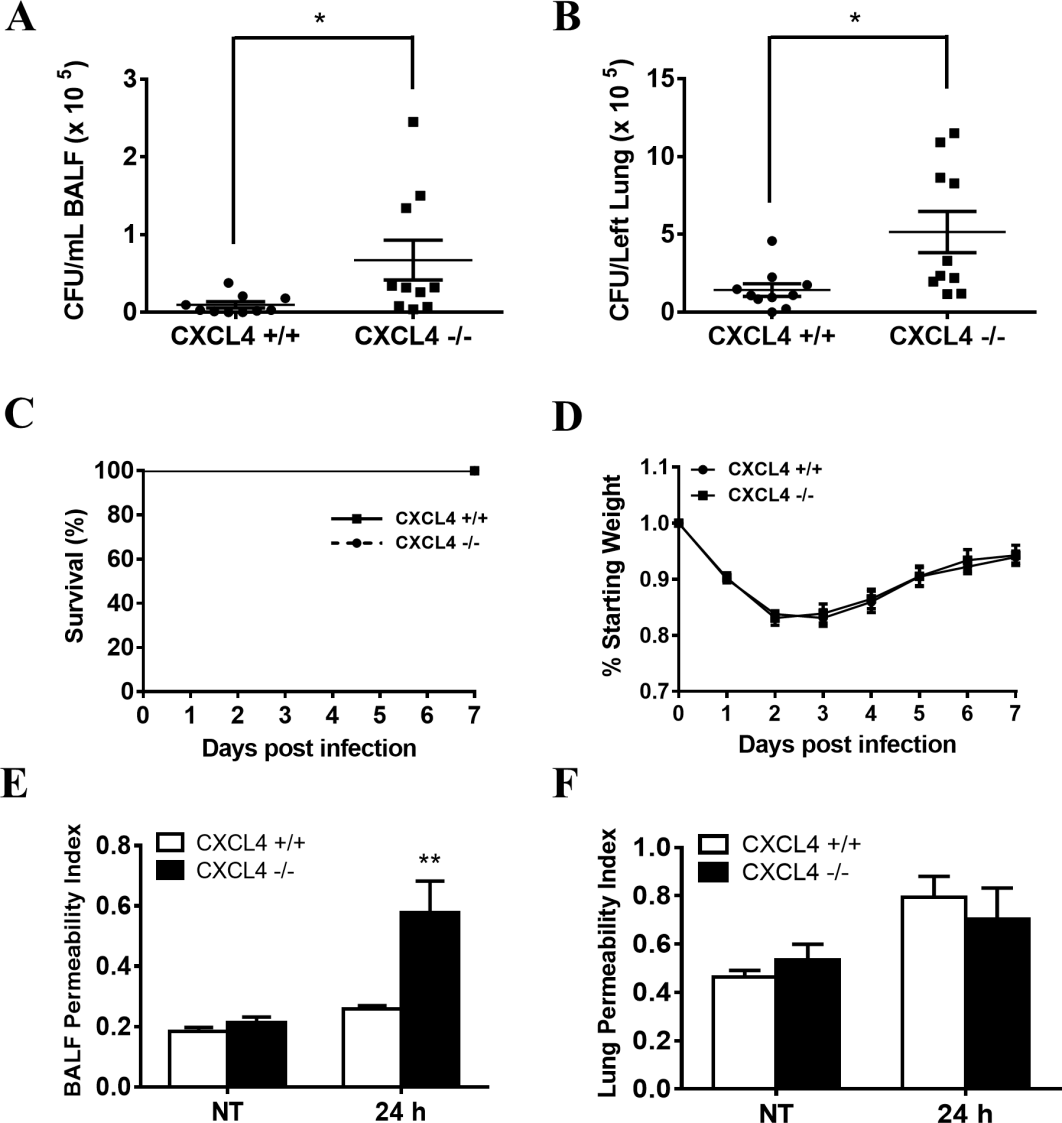
Chemokine (C-X-C motif) ligand 4 (CXCL4) deficiency impairs bacterial clearance and airway epithelial permeability after *P*. *aeruginosa* lung infection. (A, B, C, D) CXCL4 deficiency results in impaired bacterial clearance following *P*. *aeruginosa* lung infection but it does not affect host mortality and morbidity. Wild-type and CXCL4^-/-^ mice were intranasally infected with 1×10^9^ CFU of *P*. *aeruginosa* strain 8821 for 24 h. BALF and lungs were collected at 24 hpi. Serial dilutions of homogenized BALF and lung tissue were streaked onto LB agar plates and incubated 24 h at 37°C. The resulting colonies were counted to determine bacteria load (n = 9 ± SEM, *p < 0.05). For the survival study, wild-type and CXCL4^-/-^ mice were intranasally infected with 1×10^9^ CFU of *P*. *aeruginosa* strain 8821. Animal survival and body weight were monitored up to 7 days post infection (n = 12 ± SEM). (E, F) CXCL4-deficient mice display increased airway epithelial permeability after *P*. *aeruginosa* infection. Wild-type and CXCL4^-/-^ mice were left uninfected (NT) or intranasally infected with 1×10^9^ CFU of *P*. *aeruginosa* strain 8821. Six hours after infection, the mice received intraperitoneal injections of Evans blue dye. Blood, lungs and BALF were collected at 24 hpi. Dye leakage into the BALF and lungs was assessed and is presented as the OD620 ratio to a 1:20 dilution of serum (n = 4 ± SEM, **p < 0.01).

To determine the biological role of CXCL4 in *P*. *aeruginosa* infection *in vivo*, CXCL4-deficient and wild-type mice were intranasally infected with *P*. *aeruginosa* strain 8821. The bacterial burden was assessed in the lungs and BALF of CXCL4-deficient and wild-type mice at 24 hpi by CFU counting. Significantly increased number of bacteria were detected in the BALF (Fig 2A) and lungs (Fig 2B) of CXCL4-deficient mice compared with wild-type mice, suggesting that CXCL4-deficient mice have lower capacity of bacterial clearance during *P*. *aeruginosa* lung infection. Because of less cytotoxic activity of *P*. *aeruginosa* strain 8821, no mortality was observed in both CXCL4-deficient mice and wild-type mice (Fig 2C), and their body weight loss were similar (Fig 2D).

The poor clearance of *P*. *aeruginosa* in lung causes increased epithelial permeability[37]. To examine whether CXCL4 deficiency affects airway epithelial integrity, we applied the assay for analyzing Evans blue dye leakage into the airway. The BALF permeability index of CXCL4-deficient mice was markedly increased compared with wild-type mice at 24 hpi (Fig 2E). However, CXCL4-deficiency has no effect on lung permeability (Fig 2F). In addition, excessive inflammation results in increased epithelial permeability. However, CXCL4 deficiency does not affect proinflammatory cytokine responses following *P*. *aeruginosa* lung infection (Figure A-F in S4 Fig). These results suggest that CXCL4 play a protective role in airway epithelial integrity during *P*. *aeruginosa* lung infection.

### 3. CXCL4 deficiency impairs neutrophil recruitment with selectively regulating production of neutrophil recruiting chemokine after *P*. *aeruginosa* lung infection

**Fig 3:**
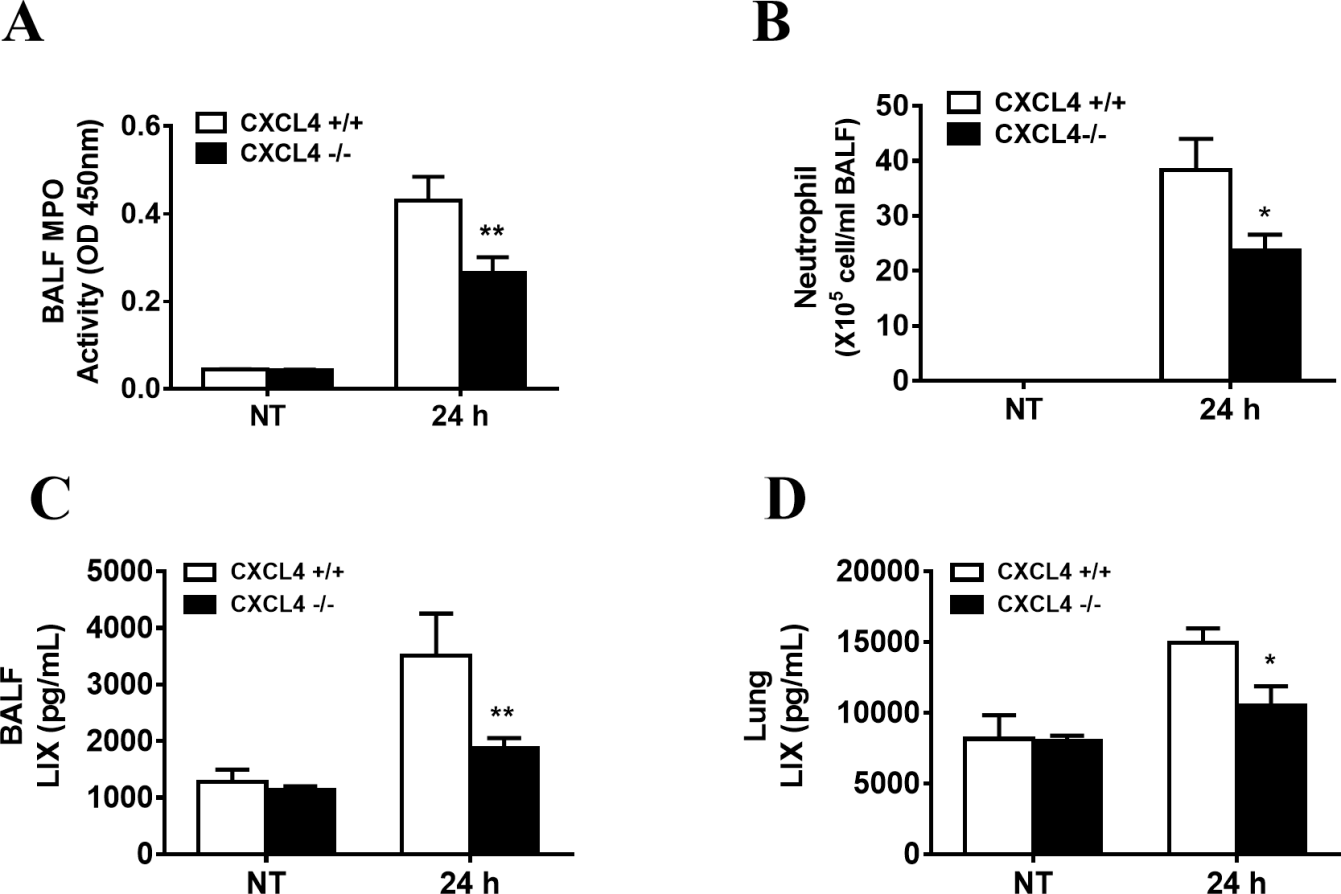
CXCL4 deficiency impairs neutrophil recruitment with regulating production of LIX after *P*. *aeruginosa* lung infection. (A, B) Wild-type and CXCL4^-/-^ mice were intranasally infected with 1×10^9^ CFU of *P*. *aeruginosa* strain 8821 or an equivalent volume of saline (NT). BALF and lungs were collected 24 hpi. Neutrophil recruitment to the BALF were assessed by MPO activity (n = 9 ± SEM, **p < 0.01) and Flow Cytometry (n = 4 ± SEM, *p < 0.05). (C-D) Supernatants of the BALF and lung homogenate were analyzed for production of LIX (n = 8 ± SEM, *p < 0.05, **p < 0.01).

Neutrophils contribute to the clearance of *P*. *aeruginosa* from the lungs[38]. We further measured the infiltration of neutrophils in lung after *P*. *aeruginosa* infection using myeloperoxidase (MPO) as a neutrophil marker. Decreased MPO levels were observed in the BALF of CXCL4-deficient mice at 24 h after *P*. *aeruginosa* infection (Fig 3A). Consistent with the MPO results, flow cytometry data also showed decreased neutrophil recruitment in the BALF of CXCL4-deficient mice (Fig 3B). We also analyzed other inflammatory cells, such as macrophages and platelets, and no significant differences were observed (Figure A-B in S5 Fig). In contrast to BALF MPO activities, the lung MPO activities in wild-type and CXCL4-deicient mice showed no significant difference at 24 hpi (Figure C in S5 Fig). The data from the histological study of the lungs were consistent with the MPO results (Figure D in S5 Fig), showing similar inflammatory cells in the lung parenchyma of CXCL4-deficient mice at 24 hpi and wild-type mice.

LIX, KC and MIP2 are murine neutrophil chemoattractant chemokines. In this study, the production of LIX (Fig 3C and 3D) was decreased in BALF and lung of CXCL4-deficient mice after *P*. *aeruginosa* infection at 24 h. No significant decrease was observed in the KC (Figure A-B in S6 Fig) and MIP2 (Figure C-D in S6 Fig) level in CXCL4-deficient mice compared to wild-type mice. This finding suggests that CXCL4 selectively regulated production of neutrophil attracting chemokines.

### 4. CXCL4 deficiency impairs platelet-neutrophil interactions in the blood following *P*. *aeruginosa* lung infection

**Fig 4:**
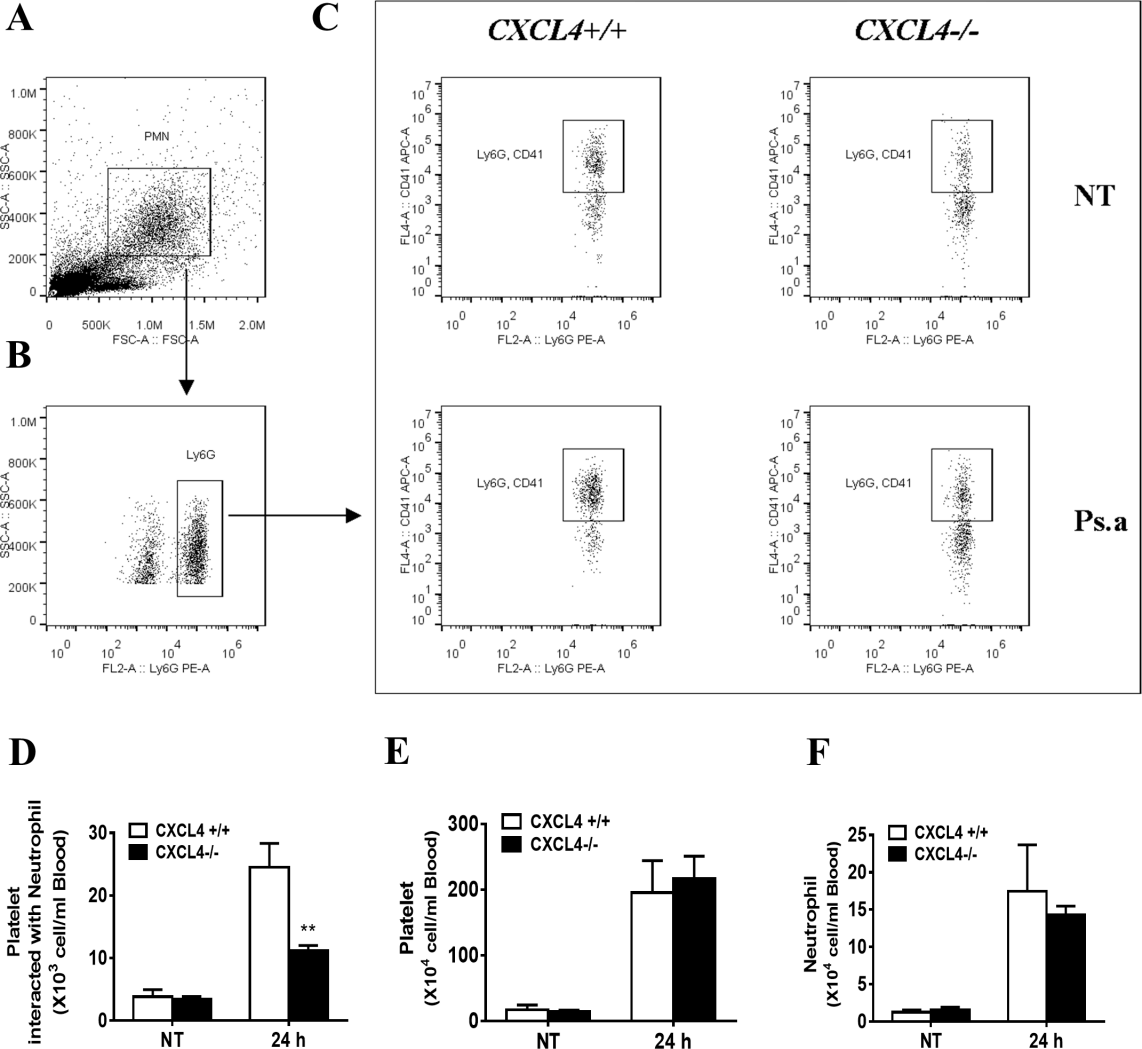
CXCL4 deficiency reduces platelet-neutrophil interactions without affecting platelet or neutrophil number in blood following *P*. *aeruginosa* lung infection. Wild-type and CXCL4^-/-^ mice were intranasally infected with 1×10^9^ CFU of *P*. *aeruginosa* strain 8821 or an equivalent volume of saline (NT). Blood were collected at 24 hpi. Red blood cells were lysed with lysis buffer. The cells were stained with Ly6G (neutrophils) and CD41 (platelets). The Ly6G^+^ CD41^+^ cell population was gated as illustrated (A, B) and the platelets interacted with neutrophils (C, D) was analyzed by flow cytometry. The total numbers of platelet and neutrophil were also counted (E, F) (n = 4 ± SEM, **p < 0.01).

Neutrophils in the circulating blood need to cross the endothelium of the blood vessels for recruitment to the lungs and BALF. Platelets play a critical role in this process via interactions with neutrophils[39]. To determine the biological role of CXCL4 in platelet-neutrophil interactions following *P*. *aeruginosa* infection *in vivo*, CXCL4-deficient and wild-type mice were intranasally infected with *P*. *aeruginosa* strain 8821. Blood was collected, and whole blood cells were isolated for flow cytometry analysis. We found that the absence of CXCL4 significantly inhibited the combination of platelets and neutrophils in the blood (Fig 4A–4E) without affecting the number of platelets or neutrophils (Fig 4F and 4G). These findings suggest that CXCL4 is critical for platelet-neutrophil interactions in the blood following *P*. *aeruginosa* lung infection.

### 5. CXCL4 contributes to the *P*. *aeruginosa*-killing capability of neutrophils

**Fig 5:**
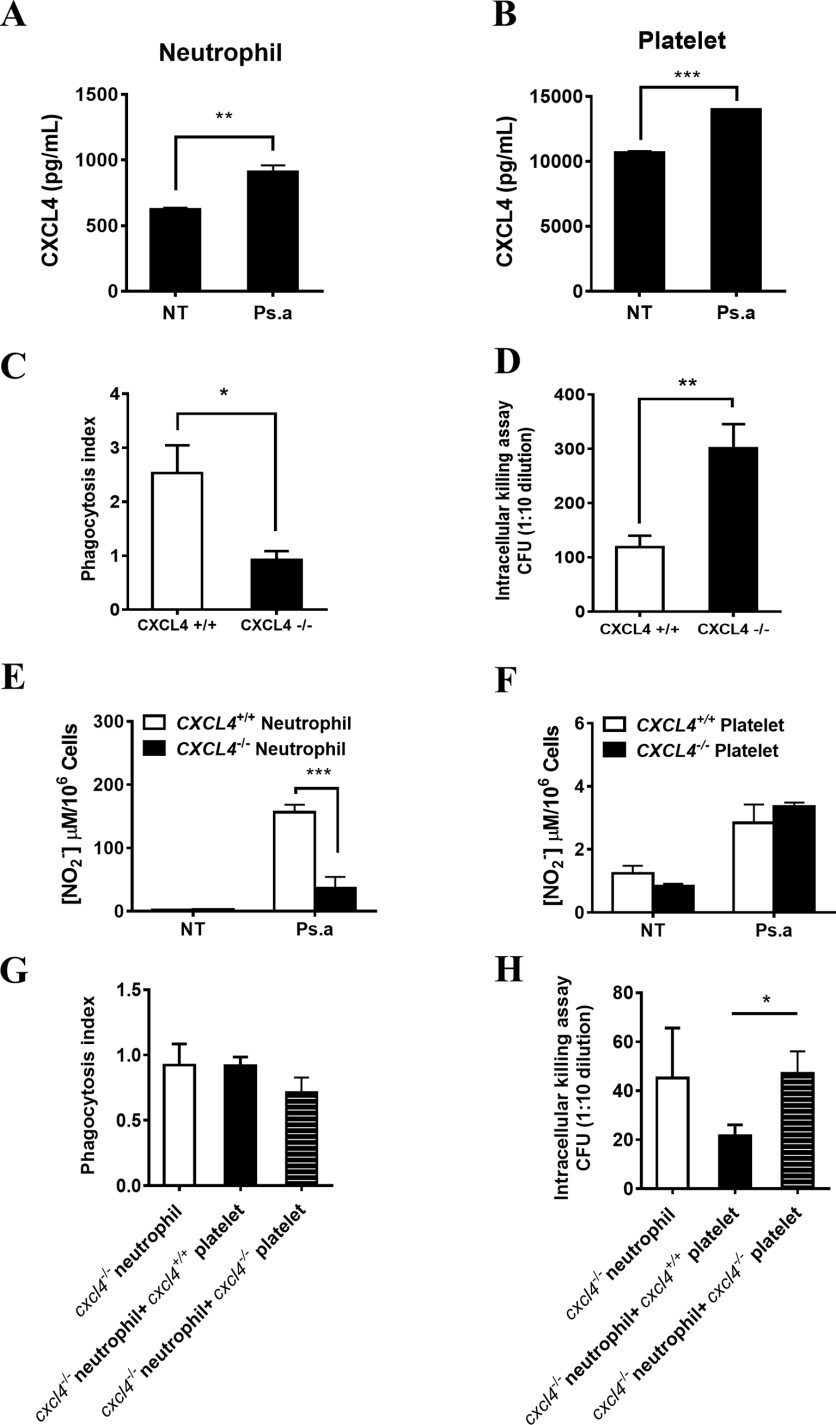
CXCL4 contributes to the *P*. *aeruginosa*-killing capability of neutrophils. (A, B) The CXCL4 levels in neutrophils and platelets was evaluated after *P*. *aeruginosa* infection *in vitro*. Wild-type bone marrow-isolated neutrophils and blood isolated-platelets were left untreated (NT) or exposed to *P*. *aeruginosa* strain 8821 at a MOI of 1:10 for 6 h. Supernatants were subjected to ELISA analysis for determining CXCL4 production (n = 3 ± SEM, **p < 0.01, ***p < 0.001). (C) The phagocytosis index was significantly decreased in CXCL4-deficient neutrophil. Mouse bone marrow-derived neutrophils were isolated from WT or CXCL4^*-/-*^ mice, and then incubated with *P*. *aeruginosa* 8821 (opsonized with mouse serum) at 37°C for 30 min. The cells were prepared using Cytospin. The specimens were then stained with Diff-Quik and examined under a microscope. The number of bacteria engulfed by 100 randomly selected neutrophils was counted. The phagocytic activity was measured according to the phagocytosis index (n = 3 ± SEM, *p<0.05). (D) Bacterial killing capacity was impaired in CXCL4-deficient neutrophil. Mouse bone marrow-derived neutrophils were isolated from WT or CXCL4^*-/-*^ mice and incubated with *P*. *aeruginosa* 8821 (opsonized with mouse serum) at 37°C for 1 h. Gentamycin was added to kill extracellular bacteria for 3 h. Then, the neutrophils were washed and lysed with PBS containing 0.1% Triton X-100. The samples were serially diluted and spread onto Luria broth (LB) agar plates. Colony numbers were determined after overnight incubation at 37°C (n = 3 ± SEM, **p<0.01). (E, F) Wild-type and CXCL4^-/-^ bone marrow isolated neutrophil or blood isolated platelet were left untreated (NT) or exposed to *P*. *aeruginosa* strain 8821 at a MOI of 1:10. Supernatants were collected at 6h and analyzed for NO production (n = 3 ± SEM, ***p < 0.001). (G, H) platelet-derived CXCL4 may facilitate the neutrophil-mediated clearance of *P*. *aeruginosa*. Mouse bone marrow-derived neutrophils and blood-derived platelets were isolated from WT or CXCL4-deficient mice. An *in vitro* phagocytosis assay and intracellular killing assay were performed as described above. (n = 3 ± SEM, *p<0.05).

Neutrophils that have been recruited to the lungs play leading roles in clearing *P*. *aeruginosa* through phagocytosis and killing[40]. The CXCL4 levels in neutrophils and platelets was evaluated after *P*. *aeruginosa* infection *in vitro* (Fig 5A and 5B). To examine whether CXCL4 affects neutrophil function, bone marrow-isolated neutrophils from wild-type mice or CXCL4-deficient mice were infected with *P*. *aeruginosa in vitro*. The phagocytosis index was significantly decreased in CXCL4-deficient neutrophil (Fig 5C), and the bacterial killing capacity was also impaired (Fig 5D). Nitric oxide (NO) mediates bacterial clearance by neutrophils. NO production was inhibited in CXCL4-deficient neutrophils but not in CXCL4-deficient platelets compared to wild-type platelets after *P*. *aeruginosa* infection (Fig 5E and 5F). In addition, when CXCL4-deficient platelets were added to CXCL4-deficient neutrophils, the phagocytosis index was decreased. However, there was no statistical significance was observed (Fig 5G). In the presence of platelets, neutrophils were more effective in killing bacteria, and this effect was blocked when CXCL4 was absent in platelets (Fig 5H). These results suggest that platelet-derived CXCL4 may facilitate the neutrophil-mediated clearance of *P*. *aeruginosa*.

## Discussion

The lungs have hematopoietic function; thus, they may function as an essential site of thrombopoiesis. Platelets have shown important immune regulatory functions, and they participate in the pulmonary inflammatory responses through circulation in pulmonary capillaries[41]. Moreover, experimental and clinical evidence have shown that platelets are also important effector cells in various lung diseases[7, 42]. Chronic or acute *P*. *aeruginosa* lung infection is a common cause of morbidity and mortality among CF patients and immunocompromised individuals. Multiple immune effector cells are involved in the host defense against *P*. *aeruginosa* lung infection via unique mechanisms. Previously, we demonstrated essential roles of macrophages[29], mast cells[43] and dendritic cells[44] in an acute *P*. *aeruginosa* infection model. Although recent studies have shown that the absence of platelets impairs host defense against chronic *P*. *aeruginosa* infection[45], the immune regulatory mechanisms of platelets are not fully understood. In this study, we identified the critical role of platelets in host defense against *P*. *aeruginosa* infection in an acute infection model. In contrast to chronic infection[45], the platelets were activated in acute infection site, and the number of platelets in blood circulation was also significantly increased during *P*. *aeruginosa* lung infection. This effect might be explained by the damaged integrity of airway epithelial permeability following a high bacterial load during acute infection. Importantly, consistent with chronic infection, elevated CXCL4 at the site of infection were observed. These findings demonstrate the contribution of platelets in response to *P*. *aeruginosa* lung infections *in vivo*, implying CXCL4 may play potential roles in the clearance of *P*. *aeruginosa*.

CXCL4 is mainly produced by megakaryocytes and platelets, with low expression in other cells such as glial cells, macrophages, and T cells. CXCL4 can block platelet aggregation, inhibit angiogenesis, and induce inflammatory responses[46]. In particular, CXCL4 can recruit neutrophils, monocytes, fibroblasts, and T cells to the sites of inflammation[47]. In addition to participating in cardiovascular diseases[48], tumors[18], systemic sclerosis[17], pancreatitis[49] and hepatic fibrosis[50], CXCL4 plays important roles in lung inflammatory diseases, such as in acute lung injury[42], infection-induced lung injury[36] and lung cancer[51], by promoting platelet production and regulating neutrophil infiltration. A variety of chemokines, including MIP-2[52], KC[53], and LIX[54], are produced during *P*. *aeruginosa* infection. However, the role of platelet-derived CXCL4 in *P*. *aeruginosa* infection remains unclear. Using CXCL4-deficient mice in our acute infection model, we elucidated the role of CXCL4 in pulmonary *P*. *aeruginosa* infection for the first time. Depletion of CXCL4 affects the binding of platelets to neutrophils in blood circulation and transmembrane migration of neutrophils to BALF, and eventually hampers *P*. *aeruginosa* clearance.

Platelets is able to maintain the basal barrier integrity of alveolar capillaries and selectively control the migration of proteins and leukocytes from vessels to lung tissues[55]. In this study, the integrity of the lungs was damaged in CXCL4-deficient mice during *P*. *aeruginosa* infection, accompanied by decreased neutrophil recruitment to the BALF, suggesting that platelet-specific CXCL4 contribute to the neutrophil infiltration in lungs. However, our results appear to conflict with the acid aspiration-induced acute lung injury (ALI) model, in which CXCL4^-/-^ mice are protected through improved barrier function without affecting neutrophil transmigration to the airways[42]. These differences can be explained by the fact that acid may have different effects on the lungs than those observed during infection with pathogens.

Activated platelets and platelet-derived microparticles can bind to leukocytes, resulting in local release of platelet-derived cytokines or chemokines. These platelet-specific factors modulate leukocyte functions and contribute to immune responses to infection[39]. CXCL4 is stored in platelet a-granules and released upon activation. Our results showed that CXCL4 deficiency reduces platelet-neutrophil interactions in blood following *P*. *aeruginosa* lung infection. Moreover, previous studies have demonstrated that platelets directly interact with neutrophils and enhance the phagocytosis of various bacteria[56, 57], thereby contributing to bacterial clearance. Interestingly, we also found that the phagocytosis index was significantly decreased when CXCL4 was absent. These results suggest that CXCL4 plays critical roles in bacterial clearance against *P*. *aeruginosa* lung infection. Future work will focus on investigation of the mechanisms involved in CXCL4-regulated platelet-neutrophil interactions in the lungs.

LIX, KC and MIP2 are important for neutrophil recruitment into rodents’ lungs via CXCR2[42]. CXCL4 affects neutrophils transmigration to the airways, but itself has no chemotactic activity for neutrophils[46]. A possible explanation is that CXCL4 activates the mouse LIX/KC/MIP-2-CXCR2 neutrophil chemotaxis pathway, as observed in influenza infection[58]. Another possible explanation is that multiple chemokines cooperate in response to pathogen infections. For example, previous studies in acute lung injury models have shown that CCL5 can form heterogeneous dimers with CXCL4 to enhance its ability to recruit monocytes[59]. In our study, decreased neutrophil recruitment to BALF was observed in CXCL4-deficient mice during *P*. *aeruginosa* infection. Interestingly, the production of LIX was also decreased in CXCL4-deficient mice, whereas no significant change in the KC and MIP2 levels. These results suggest that CXCL4 is required for specific chemokines production which regulate neutrophil recruitment, and the mechanisms should be focused in future work.

Furthermore, absence of CXCL4 did not cause a change in mortality or lead to severe lung inflammation and pathological damage, suggesting that the immunoregulatory effect of CXCL4 in *P*. *aeruginosa* infection was mild. These findings differ from those observed in respiratory virus infection in CXCL4^-/-^ mice [58]. A possible explanation is that the immune mechanisms involved in the host responses differ from the infections caused by bacteria and viruses although their infection route is similar. Furthermore, CXCL4 has been found to play a role in bacterial host defense by inducing a humoral immune response to CXCL4-coated bacteria[60]. Negatively charged lipopolysaccharide (LPS) is the CXCL4 binding structure on Gram-negative bacteria[22]. *In vitro* experiments in our study showed that platelet-derived CXCL4 contributes to the removal of *P*. *aeruginosa*. Previous studies have shown that CXCL4 receptors are expressed on the surface of neutrophils. Once activated, these receptors may mediate the bacteria-killing effect of cells via different signaling pathways[24]. In addition, neutrophils themselves can produce low levels of CXCL4. As confirmed in the present experiments, CXCL4 can also exert its effect in a platelet-independent manner. Therefore, CXCL4 is an important factor in controlling the bactericidal function of neutrophils.

Taken together with the observation of CXCL4 in protecting against *P*. *aeruginosa*-induced acute lung damage, these findings reveal a novel role of CXCL4 in fighting against *P*. *aeruginosa* lung infection and support the mechanism by which platelet-derived CXCL4 affects neutrophil recruitment and bacteria-killing functions. Moreover, these findings expand our knowledge on divergent roles of CXCL4 in response to various stimuli and highlight the critical role of platelets in mediation of CXCL4 production during *P*. *aeruginosa* lung infection. During *P*. *aeruginosa* infection, CXCL4 has a specific regulatory effect on neutrophil recruitment and function, accompanied with a relatively mild pathogenic effect. Therefore, CXCL4 may be a potential therapeutic target in inflammatory diseases.

## Supporting information

S1 Fig Anti-platelet serum depletes circulating platelet in *P*. *aeruginosa* infected mice. Sixteen hours prior to infection with *P*. *aeruginosa*, wild-type mice were administered an intraperitoneal injection of 50 μL of rabbit anti-mouse platelet serum and control serum. Control and platelet-depleted mice were intranasally infected with 1×10^9^ CFU of *P*. *aeruginosa* strain 8821. Whole blood cells were collected at 24 hpi. The depletion of the platelet was confirmed by flow cytometry (A). Serum supernatants were collected for determining CXCL4 production by ELISA (B) (n = 3–4 ± SEM, **p < 0.01).
(TIF)

S2 Fig Platelet depletion causes severe lung damage following *P*. *aeruginosa* lung infection. Wild-type and platelet depleted mice were infected intranasally with 1×10^9^ CFU of *P*. *aeruginosa* strain 8821 or an equivalent volume of saline (NT) for 24 hours later. After infection, the upper lobe of the left lung was collected for H&E staining (original magnification × 100 for panels a-d).
(TIF)

S3 Fig Platelet depletion does not impact BALF and blood bacterial clearance following *P*. *aeruginosa* lung infection. Sixteen hours before infection with *P*. *aeruginosa*, an intraperitoneal injection of 50 μL of rabbit anti-mouse platelet serum was performed on wild-type mice. Untreated wild-type and platelet depleted mice were infected intranasally with 1×10^9^ CFU of *P*. *aeruginosa* strain 8821 for 24 hours. BALF and blood were collected at 24 hpi. Serial dilution of homogenized BALF (A) and blood (B) was streaked on LB agar plates and incubated 24 h at 37°C. The resultant colonies were counted to determine bacterial burden (n = 9 ± SEM).
(TIF)

S4 Fig CXCL4 deficiency has no effect on proinflammatory cytokine production following *P*. *aeruginosa* lung infection. Wild-type and CXCL4^-/-^ mice were infected intranasally with 1×10^9^ CFU of *P*. *aeruginosa* strain 8821 or an equivalent volume of saline (NT). Lung and BALF were collected 24 hpi. Supernatants were subjected to ELISA analysis for proinflammatory cytokine TNF (A, B), IL-6 (C, D) and IL-1β (E, F) (n = 8 ± SEM).
(TIF)

S5 Fig CXCL4 deficient mice display limit effect on infiltration of inflammatory cells to BALF and lung after *P*. *aeruginosa* infection. Wild-type and CXCL4^-/-^ mice were infected intranasally with 1×10^9^ CFU of *P*. *aeruginosa* strain 8821 or an equivalent volume of saline (NT). BALF and Lung were collected 24 hpi. Macrophage (A) and platelet (B) recruitment to the BALF was assessed by Flow Cytometry. Neutrophil recruitment to the lung (C) were assessed by MPO activity. The upper lobe of the left lung was collected for H&E staining (D, original magnification × 200 for panels a-d) (n = 9 ± SEM).
(TIF)

S6 Fig No significant decrease was observed in the KC and MIP2 level in CXCL4-deficient mice compared to wild-type mice. Wild-type and CXCL4^-/-^ mice were infected intranasal with 1×10^9^ CFU of *P*. *aeruginosa* strain 8821 or an equivalent volume of saline (NT). Lung and BALF were collected 24 hpi. Supernatants of BALF and lung homogenate were analyzed for production of KC and MIP2 (n = 8 ± SEM).
(TIF)

## References

[1] Cripps AW, Dunkley ML, Clancy RL, Kyd J. Pulmonary immunity to Pseudomonas aeruginosa. Immunology and cell biology. 1995;73(5):418–24. 10.1038/icb.1995.65 . 8595919

[2] Speert DP. Phagocytosis of Pseudomonas aeruginosa by macrophages: receptor-ligand interactions. Trends in microbiology. 1993;1(6):217–21. . 8137118 10.1016/0966-842x(93)90135-e

[3] Wesselkamper SC, Eppert BL, Motz GT, Lau GW, Hassett DJ, Borchers MT. NKG2D is critical for NK cell activation in host defense against Pseudomonas aeruginosa respiratory infection. Journal of immunology. 2008;181(8):5481–9. ; PubMed Central PMCID: PMC2567053. 18832705 10.4049/jimmunol.181.8.5481 PMC2567053

[4] Powderly WG, Pier GB, Markham RB. T lymphocyte-mediated protection against Pseudomonas aeruginosa infection in granulocytopenic mice. The Journal of clinical investigation. 1986;78(2):375–80. 10.1172/JCI112587 ; PubMed Central PMCID: PMC423557. 2426306 PMC423557

[5] Ritchie AJ, Yam AO, Tanabe KM, Rice SA, Cooley MA. Modification of in vivo and in vitro T- and B-cell-mediated immune responses by the Pseudomonas aeruginosa quorum-sensing molecule N-(3-oxododecanoyl)-L-homoserine lactone. Infection and immunity. 2003;71(8):4421–31. 10.1128/IAI.71.8.4421-4431.2003 ; PubMed Central PMCID: PMC165988. 12874321 PMC165988

[6] Golebiewska EM, Poole AW. Platelet secretion: From haemostasis to wound healing and beyond. Blood Rev. 2015;29(3):153–62. 10.1016/j.blre.2014.10.003 ; PubMed Central PMCID: PMCPMC4452143. 25468720 PMC4452143

[7] Page C, Pitchford S. Platelets and allergic inflammation. Clinical and experimental allergy: journal of the British Society for Allergy and Clinical Immunology. 2014;44(7):901–13. 10.1111/cea.12322 . 24708345

[8] Mantovani A, Garlanda C. Platelet-macrophage partnership in innate immunity and inflammation. Nature immunology. 2013;14(8):768–70. 10.1038/ni.2666 . 23867924

[9] Yeaman MR. Bacterial-platelet interactions: virulence meets host defense. Future microbiology. 2010;5(3):471–506. 10.2217/fmb.09.112 . 20210555

[10] Kerrigan SW. The expanding field of platelet-bacterial interconnections. Platelets. 2015;26(4):293–301. 10.3109/09537104.2014.997690 . 25734214

[11] Coutinho IR, Berk RS, Mammen E. Platelet aggregation by a phospholipase C from Pseudomonas aeruginosa. Thrombosis research. 1988;51(5):495–505. . 3140410 10.1016/0049-3848(88)90115-6

[12] Barbier M, Owings JP, Martinez-Ramos I, Damron FH, Gomila R, Blazquez J, et al Lysine trimethylation of EF-Tu mimics platelet-activating factor to initiate Pseudomonas aeruginosa pneumonia. mBio. 2013;4(3):e00207–13. 10.1128/mBio.00207-13 ; PubMed Central PMCID: PMC3663188. 23653444 PMC3663188

[13] Machado GB, de Assis MC, Leao R, Saliba AM, Silva MC, Suassuna JH, et al ExoU-induced vascular hyperpermeability and platelet activation in the course of experimental Pseudomonas aeruginosa pneumosepsis. Shock. 2010;33(3):315–21. 10.1097/SHK.0b013e3181b2b0f4 . 19543153

[14] Lasagni L, Francalanci M, Annunziato F, Lazzeri E, Giannini S, Cosmi L, et al An alternatively spliced variant of CXCR3 mediates the inhibition of endothelial cell growth induced by IP-10, Mig, and I-TAC, and acts as functional receptor for platelet factor 4. The Journal of experimental medicine. 2003;197(11):1537–49. 10.1084/jem.20021897 ; PubMed Central PMCID: PMC2193908. 12782716 PMC2193908

[15] Struyf S, Salogni L, Burdick MD, Vandercappellen J, Gouwy M, Noppen S, et al Angiostatic and chemotactic activities of the CXC chemokine CXCL4L1 (platelet factor-4 variant) are mediated by CXCR3. Blood. 2011;117(2):480–8. 10.1182/blood-2009-11-253591 ; PubMed Central PMCID: PMC3031477. 20980681 PMC3031477

[16] Gleissner CA, Shaked I, Little KM, Ley K. CXC chemokine ligand 4 induces a unique transcriptome in monocyte-derived macrophages. Journal of immunology. 2010;184(9):4810–8. 10.4049/jimmunol.0901368 ; PubMed Central PMCID: PMC3418140. 20335529 PMC3418140

[17] van Bon L, Affandi AJ, Broen J, Christmann RB, Marijnissen RJ, Stawski L, et al Proteome-wide analysis and CXCL4 as a biomarker in systemic sclerosis. The New England journal of medicine. 2014;370(5):433–43. 10.1056/NEJMoa1114576 ; PubMed Central PMCID: PMC4040466. 24350901 PMC4040466

[18] Fang S, Liu B, Sun Q, Zhao J, Qi H, Li Q. Platelet factor 4 inhibits IL-17/Stat3 pathway via upregulation of SOCS3 expression in melanoma. Inflammation. 2014;37(5):1744–50. 10.1007/s10753-014-9903-4 . 24799320

[19] McMorran BJ, Wieczorski L, Drysdale KE, Chan JA, Huang HM, Smith C, et al Platelet factor 4 and Duffy antigen required for platelet killing of Plasmodium falciparum. Science. 2012;338(6112):1348–51. 10.1126/science.1228892 . 23224555

[20] Yeaman MR. Platelets in defense against bacterial pathogens. Cellular and molecular life sciences: CMLS. 2010;67(4):525–44. 10.1007/s00018-009-0210-4 ; PubMed Central PMCID: PMC2809947. 20013024 PMC2809947

[21] Wong CH, Jenne CN, Petri B, Chrobok NL, Kubes P. Nucleation of platelets with blood-borne pathogens on Kupffer cells precedes other innate immunity and contributes to bacterial clearance. Nature immunology. 2013;14(8):785–92. 10.1038/ni.2631 . 23770641 PMC4972575

[22] Krauel K, Weber C, Brandt S, Zahringer U, Mamat U, Greinacher A, et al Platelet factor 4 binding to lipid A of Gram-negative bacteria exposes PF4/heparin-like epitopes. Blood. 2012;120(16):3345–52. 10.1182/blood-2012-06-434985 . 22942185

[23] Arman M, Krauel K, Tilley DO, Weber C, Cox D, Greinacher A, et al Amplification of bacteria-induced platelet activation is triggered by FcgammaRIIA, integrin alphaIIbbeta3, and platelet factor 4. Blood. 2014;123(20):3166–74. 10.1182/blood-2013-11-540526 ; PubMed Central PMCID: PMC4023422. 24642751 PMC4023422

[24] Kasper B, Petersen F. Molecular pathways of platelet factor 4/CXCL4 signaling. European journal of cell biology. 2011;90(6–7):521–6. 10.1016/j.ejcb.2010.12.002 . 21295372

[25] Lovewell RR, Hayes SM, O'Toole GA, Berwin B. Pseudomonas aeruginosa flagellar motility activates the phagocyte PI3K/Akt pathway to induce phagocytic engulfment. American journal of physiology Lung cellular and molecular physiology. 2014;306(7):L698–707. 10.1152/ajplung.00319.2013 ; PubMed Central PMCID: PMC3962627. 24487390 PMC3962627

[26] Roy S, Karmakar M, Pearlman E. CD14 mediates Toll-like receptor 4 (TLR4) endocytosis and spleen tyrosine kinase (Syk) and interferon regulatory transcription factor 3 (IRF3) activation in epithelial cells and impairs neutrophil infiltration and Pseudomonas aeruginosa killing in vivo. The Journal of biological chemistry. 2014;289(2):1174–82. 10.1074/jbc.M113.523167 ; PubMed Central PMCID: PMC3887184. 24275652 PMC3887184

[27] Losa D, Kohler T, Bellec J, Dudez T, Crespin S, Bacchetta M, et al Pseudomonas aeruginosa-induced apoptosis in airway epithelial cells is mediated by gap junctional communication in a JNK-dependent manner. Journal of immunology. 2014;192(10):4804–12. 10.4049/jimmunol.1301294 . 24733844

[28] Vikstrom E, Magnusson KE, Pivoriunas A. The Pseudomonas aeruginosa quorum-sensing molecule N-(3-oxododecanoyl)-L-homoserine lactone stimulates phagocytic activity in human macrophages through the p38 MAPK pathway. Microbes and infection / Institut Pasteur. 2005;7(15):1512–8. 10.1016/j.micinf.2005.05.012 . 16039899

[29] Junkins RD, MacNeil AJ, Wu Z, McCormick C, Lin TJ. Regulator of calcineurin 1 suppresses inflammation during respiratory tract infections. Journal of immunology. 2013;190(10):5178–86. 10.4049/jimmunol.1203196 . 23589609

[30] O'Sullivan R, Carrigan SO, Marshall JS, Lin TJ. Signal transducer and activator of transcription 4 (STAT4), but not IL-12 contributes to Pseudomonas aeruginosa-induced lung inflammation in mice. Immunobiology. 2008;213(6):469–79. 10.1016/j.imbio.2007.11.007 . 18514749

[31] Eslin DE, Zhang C, Samuels KJ, Rauova L, Zhai L, Niewiarowski S, et al Transgenic mice studies demonstrate a role for platelet factor 4 in thrombosis: dissociation between anticoagulant and antithrombotic effect of heparin. Blood. 2004;104(10):3173–80. 10.1182/blood-2003-11-3994 . 14764524

[32] Kamath S, Kapatral V, Chakrabarty AM. Cellular function of elastase in Pseudomonas aeruginosa: role in the cleavage of nucleoside diphosphate kinase and in alginate synthesis. Molecular microbiology. 1998;30(5):933–41. . 9988471 10.1046/j.1365-2958.1998.01121.x

[33] Boudreau RT, Garduno R, Lin TJ. Protein phosphatase 2A and protein kinase Calpha are physically associated and are involved in Pseudomonas aeruginosa-induced interleukin 6 production by mast cells. The Journal of biological chemistry. 2002;277(7):5322–9. 10.1074/jbc.M108623200 . 11706031

[34] Schneider T, Issekutz AC. Quantitation of eosinophil and neutrophil infiltration into rat lung by specific assays for eosinophil peroxidase and myeloperoxidase. Application in a Brown Norway rat model of allergic pulmonary inflammation. Journal of immunological methods. 1996;198(1):1–14. . 8914592 10.1016/0022-1759(96)00143-3

[35] Gil-Bernabe AM, Ferjancic S, Tlalka M, Zhao L, Allen PD, Im JH, et al Recruitment of monocytes/macrophages by tissue factor-mediated coagulation is essential for metastatic cell survival and premetastatic niche establishment in mice. Blood. 2012;119(13):3164–75. 10.1182/blood-2011-08-376426 . 22327225

[36] Hwaiz R, Rahman M, Zhang E, Thorlacius H. Platelet secretion of CXCL4 is Rac1-dependent and regulates neutrophil infiltration and tissue damage in septic lung damage. Br J Pharmacol. 2015;172(22):5347–59. 10.1111/bph.13325 ; PubMed Central PMCID: PMCPMC5341222. 26478565 PMC5341222

[37] Azghani AO, Miller EJ, Peterson BT. Virulence factors from Pseudomonas aeruginosa increase lung epithelial permeability. Lung. 2000;178(5):261–9. . 11147311 10.1007/s004080000031

[38] Gregory AD, Hogue LA, Ferkol TW, Link DC. Regulation of systemic and local neutrophil responses by G-CSF during pulmonary Pseudomonas aeruginosa infection. Blood. 2007;109(8):3235–43. 10.1182/blood-2005-01-015081 ; PubMed Central PMCID: PMCPMC1852251. 17185469 PMC1852251

[39] Kral JB, Schrottmaier WC, Salzmann M, Assinger A. Platelet Interaction with Innate Immune Cells. Transfus Med Hemother. 2016;43(2):78–88. 10.1159/000444807 ; PubMed Central PMCID: PMCPMC4872052. 27226790 PMC4872052

[40] Rada B. Interactions between Neutrophils and Pseudomonas aeruginosa in Cystic Fibrosis. Pathogens. 2017;6(1). 10.3390/pathogens6010010 ; PubMed Central PMCID: PMCPMC5371898. 28282951 PMC5371898

[41] Weyrich AS, Zimmerman GA. Platelets in lung biology. Annu Rev Physiol. 2013;75:569–91. 10.1146/annurev-physiol-030212-183752 ; PubMed Central PMCID: PMCPMC3670819. 23043249 PMC3670819

[42] Bdeir K, Gollomp K, Stasiak M, Mei J, Papiewska-Pajak I, Zhao G, et al Platelet-Specific Chemokines Contribute to the Pathogenesis of Acute Lung Injury. Am J Respir Cell Mol Biol. 2017;56(2):261–70. 10.1165/rcmb.2015-0245OC ; PubMed Central PMCID: PMCPMC5455412. 27755915 PMC5455412

[43] Junkins RD, Carrigan SO, Wu Z, Stadnyk AW, Cowley E, Issekutz T, et al Mast cells protect against Pseudomonas aeruginosa-induced lung injury. Am J Pathol. 2014;184(8):2310–21. 10.1016/j.ajpath.2014.05.009 . 25043620

[44] Yue L, Xie Z, Li H, Pang Z, Junkins RD, Tremblay ML, et al Protein Tyrosine Phosphatase-1B Negatively Impacts Host Defense against Pseudomonas aeruginosa Infection. Am J Pathol. 2016;186(5):1234–44. 10.1016/j.ajpath.2016.01.005 . 27105736

[45] Amison RT, O'Shaughnessy BG, Arnold S, Cleary SJ, Nandi M, Pitchford SC, et al Platelet Depletion Impairs Host Defence to Pulmonary Infection with Pseudomonas aeruginosa in Mice. Am J Respir Cell Mol Biol. 2017 10.1165/rcmb.2017-0083OC . 28957635

[46] Vandercappellen J, Van Damme J, Struyf S. The role of the CXC chemokines platelet factor-4 (CXCL4/PF-4) and its variant (CXCL4L1/PF-4var) in inflammation, angiogenesis and cancer. Cytokine Growth Factor Rev. 2011;22(1):1–18. 10.1016/j.cytogfr.2010.10.011 . 21111666

[47] Bakogiannis C, Sachse M, Stamatelopoulos K, Stellos K. Platelet-derived chemokines in inflammation and atherosclerosis. Cytokine. 2017 10.1016/j.cyto.2017.09.013 . 29198385

[48] Gleissner CA. Macrophage Phenotype Modulation by CXCL4 in Atherosclerosis. Front Physiol. 2012;3:1 10.3389/fphys.2012.00001 ; PubMed Central PMCID: PMCPMC3257836. 22275902 PMC3257836

[49] Wetterholm E, Linders J, Merza M, Regner S, Thorlacius H. Platelet-derived CXCL4 regulates neutrophil infiltration and tissue damage in severe acute pancreatitis. Transl Res. 2016;176:105–18. 10.1016/j.trsl.2016.04.006 . 27183218

[50] Zaldivar MM, Pauels K, von Hundelshausen P, Berres ML, Schmitz P, Bornemann J, et al CXC chemokine ligand 4 (Cxcl4) is a platelet-derived mediator of experimental liver fibrosis. Hepatology. 2010;51(4):1345–53. 10.1002/hep.23435 . 20162727

[51] Pucci F, Rickelt S, Newton AP, Garris C, Nunes E, Evavold C, et al PF4 Promotes Platelet Production and Lung Cancer Growth. Cell Rep. 2016;17(7):1764–72. 10.1016/j.celrep.2016.10.031 ; PubMed Central PMCID: PMCPMC5108525. 27829148 PMC5108525

[52] Kernacki KA, Barrett RP, Hobden JA, Hazlett LD. Macrophage inflammatory protein-2 is a mediator of polymorphonuclear neutrophil influx in ocular bacterial infection. Journal of immunology. 2000;164(2):1037–45. . 10623854 10.4049/jimmunol.164.2.1037

[53] Bryant-Hudson KM, Carr DJ. CXCL1-deficient mice are highly sensitive to pseudomonas aeruginosa but not herpes simplex virus type 1 corneal infection. Invest Ophthalmol Vis Sci. 2012;53(11):6785–92. 10.1167/iovs.12-10400 ; PubMed Central PMCID: PMCPMC3462609. 22952118 PMC3462609

[54] Gregson AL, Wang X, Weigt SS, Palchevskiy V, Lynch JP 3rd, Ross DJ, et al Interaction between Pseudomonas and CXC chemokines increases risk of bronchiolitis obliterans syndrome and death in lung transplantation. Am J Respir Crit Care Med. 2013;187(5):518–26. 10.1164/rccm.201207-1228OC ; PubMed Central PMCID: PMCPMC3733405. 23328531 PMC3733405

[55] Deppermann C. Platelets and vascular integrity. Platelets. 2018:1–7. 10.1080/09537104.2018.1428739 . 29446689

[56] Assinger A, Laky M, Schabbauer G, Hirschl AM, Buchberger E, Binder BR, et al Efficient phagocytosis of periodontopathogens by neutrophils requires plasma factors, platelets and TLR2. Journal of thrombosis and haemostasis: JTH. 2011;9(4):799–809. 10.1111/j.1538-7836.2011.04193.x . 21251195

[57] Hurley SM, Kahn F, Nordenfelt P, Morgelin M, Sorensen OE, Shannon O. Platelet-Dependent Neutrophil Function Is Dysregulated by M Protein from Streptococcus pyogenes. Infection and immunity. 2015;83(9):3515–25. 10.1128/IAI.00508-15 ; PubMed Central PMCID: PMCPMC4534654. 26099589 PMC4534654

[58] Guo L, Feng K, Wang YC, Mei JJ, Ning RT, Zheng HW, et al Critical role of CXCL4 in the lung pathogenesis of influenza (H1N1) respiratory infection. Mucosal Immunol. 2017;10(6):1529–41. 10.1038/mi.2017.1 . 28120850

[59] Grommes J, Alard JE, Drechsler M, Wantha S, Morgelin M, Kuebler WM, et al Disruption of platelet-derived chemokine heteromers prevents neutrophil extravasation in acute lung injury. Am J Respir Crit Care Med. 2012;185(6):628–36. 10.1164/rccm.201108-1533OC ; PubMed Central PMCID: PMCPMC3326286. 22246174 PMC3326286

[60] Krauel K, Potschke C, Weber C, Kessler W, Furll B, Ittermann T, et al Platelet factor 4 binds to bacteria, [corrected] inducing antibodies cross-reacting with the major antigen in heparin-induced thrombocytopenia. Blood. 2011;117(4):1370–8. 10.1182/blood-2010-08-301424 . 20959601

