# Supplementary figures and images for: CXCL4 contributes to host defense against acute Pseudomonas aeruginosa lung infection
Source: PLoS One. 2018 Oct 8;13(10):e0205521. doi: 10.1371/journal.pone.0205521 (PMC6175521; doi:10.1371/journal.pone.0205521)

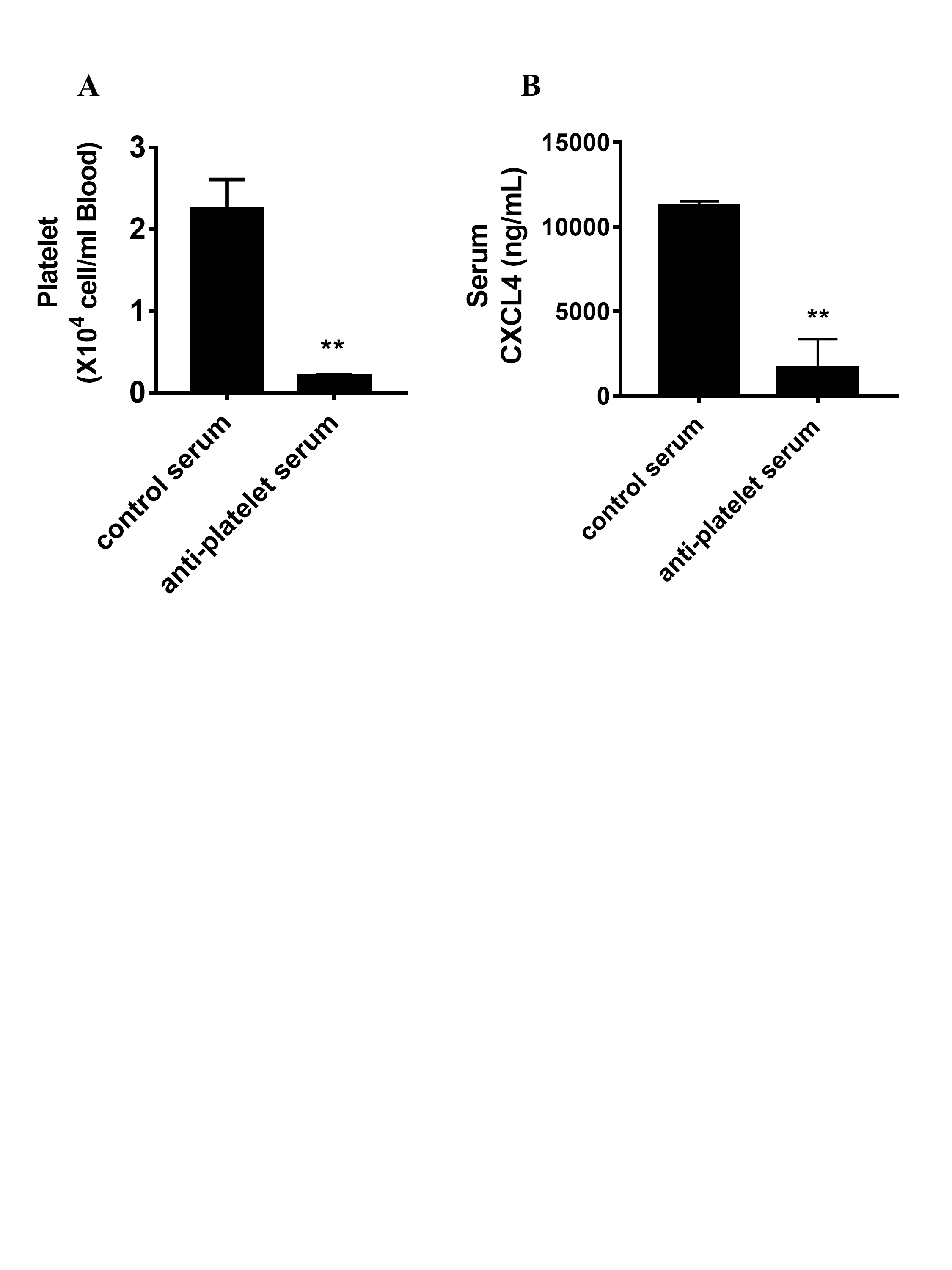

Supplement: S1 Fig — Sixteen hours prior to infection with P. aeruginosa, wild-type mice were administered an intraperitoneal injection of 50 μL of rabbit anti-mouse platelet serum and control serum. Control and platelet-depleted mice were intranasally infected with 1×10^9^ CFU of P. aeruginosa strain 8821. Whole blood cells were collected at 24 hpi. The depletion of the platelet was confirmed by flow cytometry (A). Serum supernatants were collected for determining CXCL4 production by ELISA (B) (n = 3–4 ± SEM, **p < 0.01). (TIF) [file pone.0205521.s001.TIF]

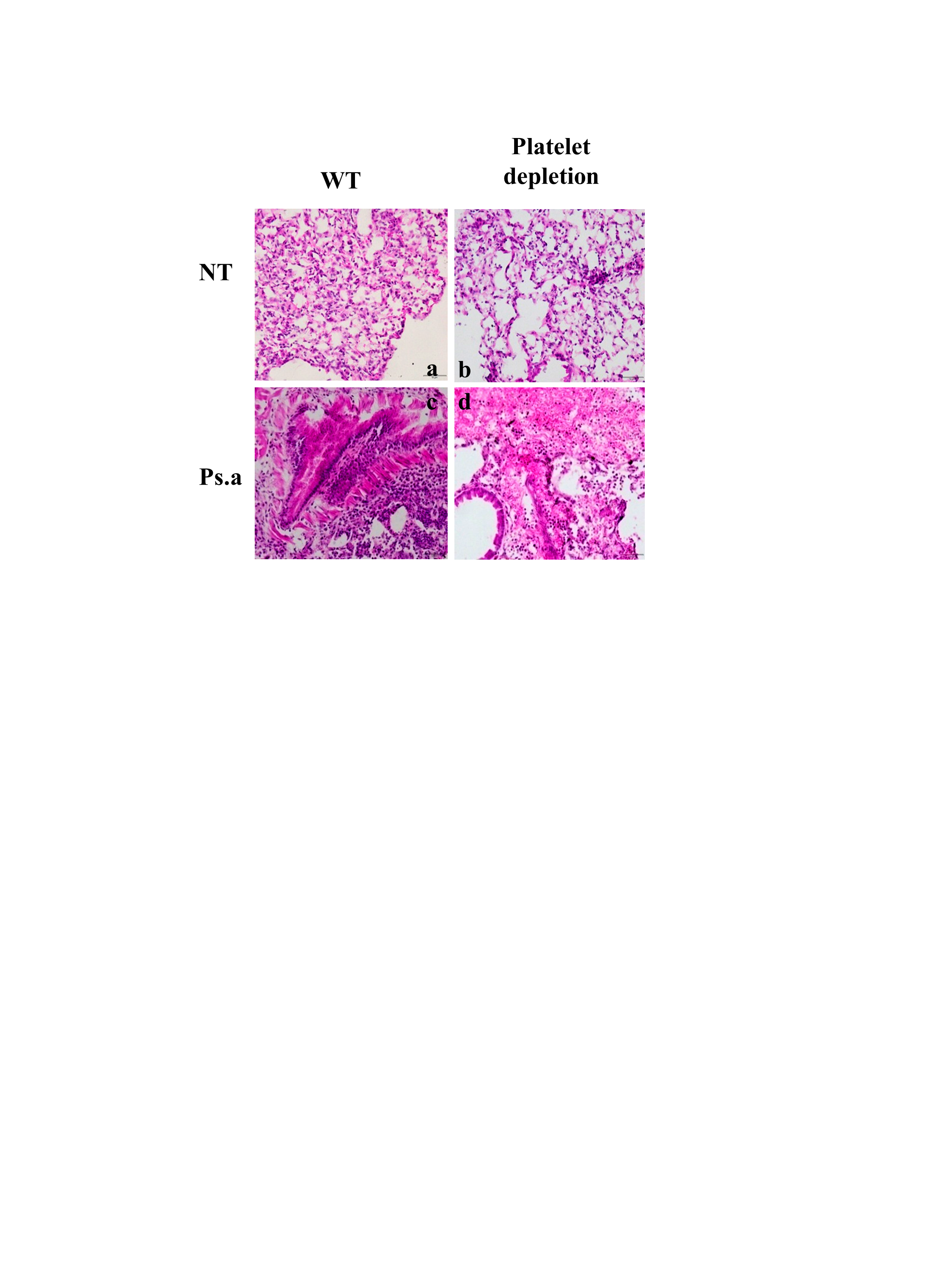

Supplement: S2 Fig — Wild-type and platelet depleted mice were infected intranasally with 1×10^9^ CFU of P. aeruginosa strain 8821 or an equivalent volume of saline (NT) for 24 hours later. After infection, the upper lobe of the left lung was collected for H&E staining (original magnification × 100 for panels a-d). (TIF) [file pone.0205521.s002.TIF]

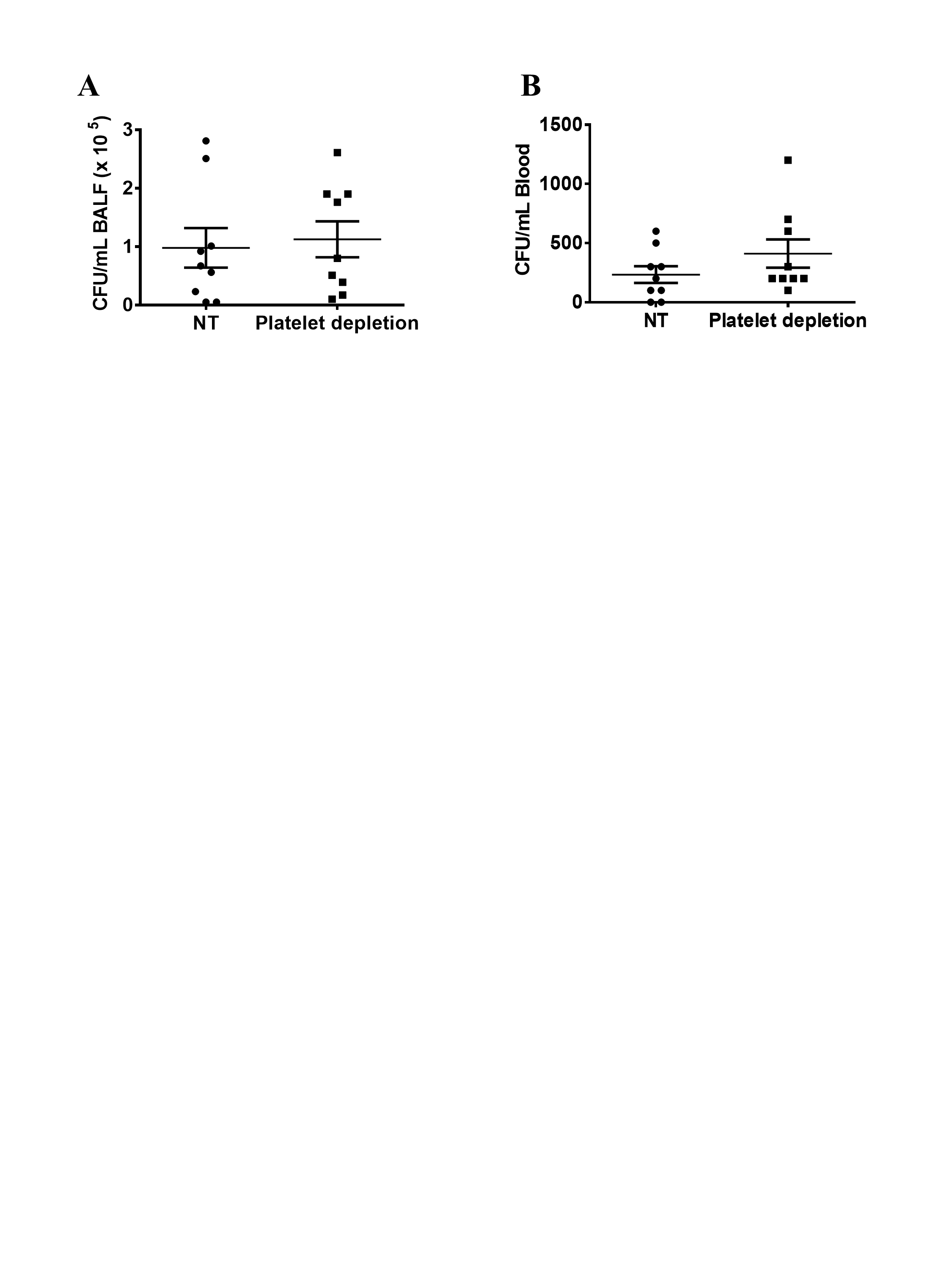

Supplement: S3 Fig — Sixteen hours before infection with P. aeruginosa, an intraperitoneal injection of 50 μL of rabbit anti-mouse platelet serum was performed on wild-type mice. Untreated wild-type and platelet depleted mice were infected intranasally with 1×10^9^ CFU of P. aeruginosa strain 8821 for 24 hours. BALF and blood were collected at 24 hpi. Serial dilution of homogenized BALF (A) and blood (B) was streaked on LB agar plates and incubated 24 h at 37°C. The resultant colonies were counted to determine bacterial burden (n = 9 ± SEM). (TIF) [file pone.0205521.s003.TIF]

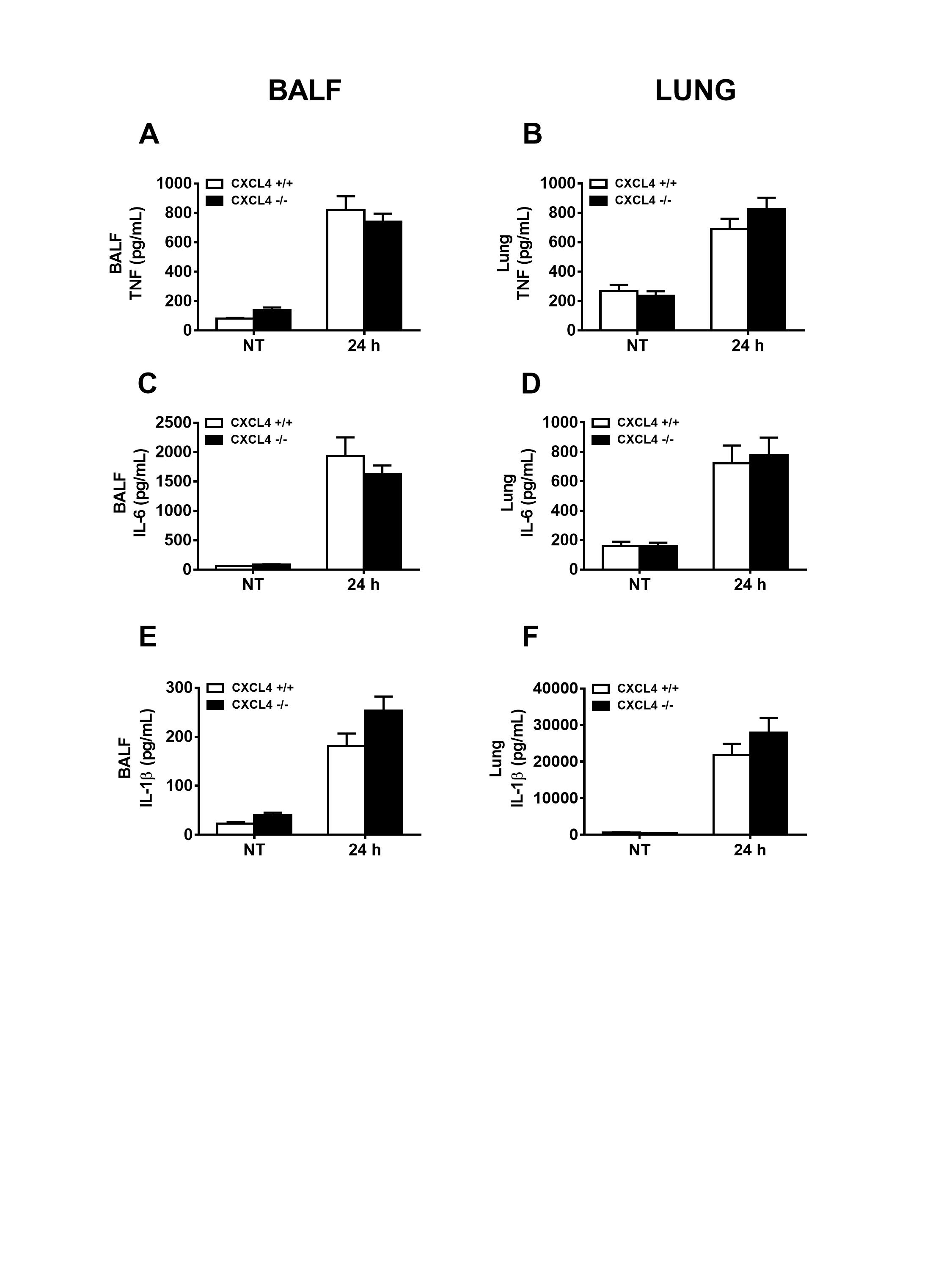

Supplement: S4 Fig — Wild-type and CXCL4-/- mice were infected intranasally with 1×10^9^ CFU of P. aeruginosa strain 8821 or an equivalent volume of saline (NT). Lung and BALF were collected 24 hpi. Supernatants were subjected to ELISA analysis for proinflammatory cytokine TNF (A, B), IL-6 (C, D) and IL-1β (E, F) (n = 8 ± SEM). (TIF) [file pone.0205521.s004.TIF]

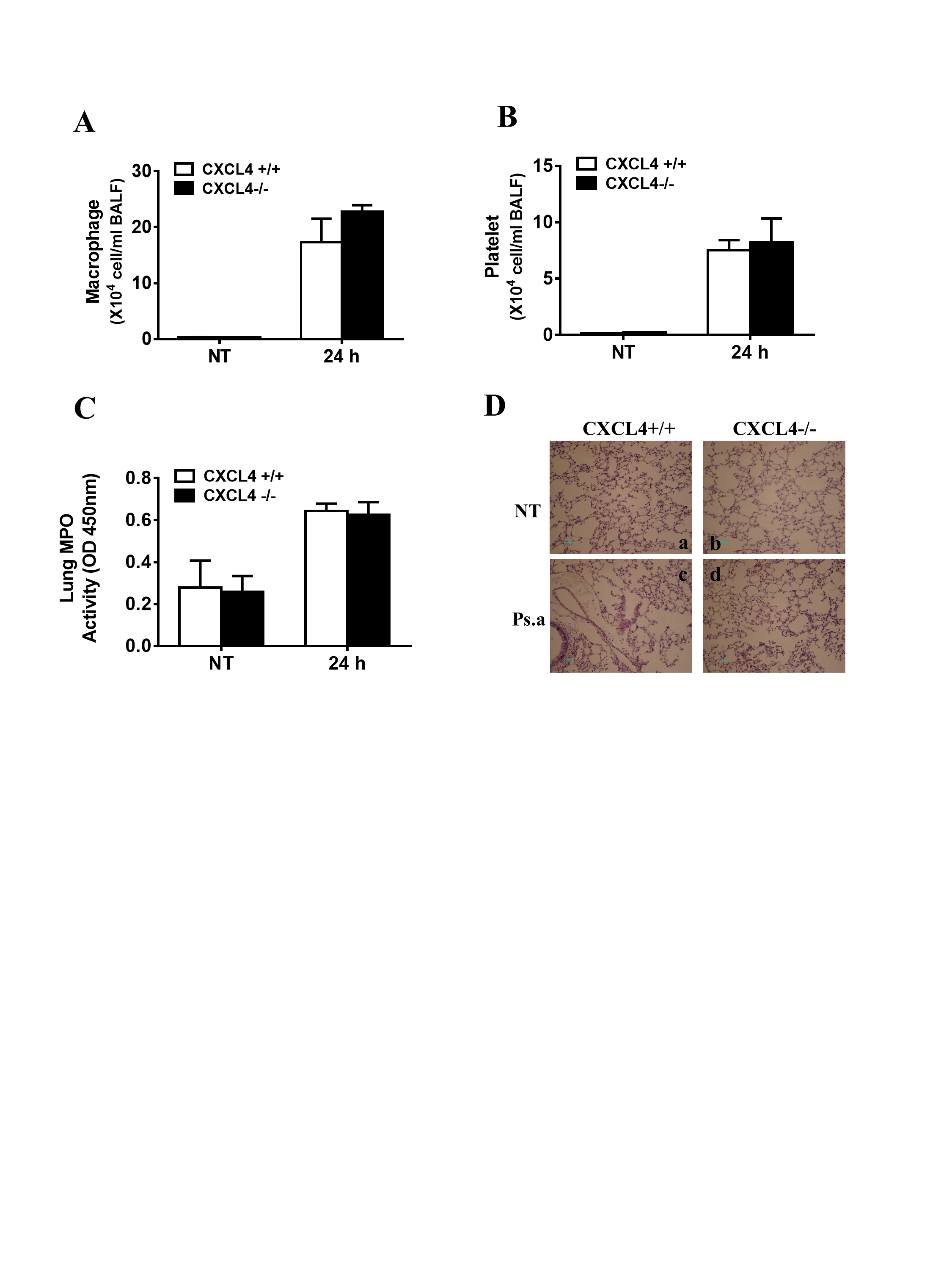

Supplement: S5 Fig — Wild-type and CXCL4-/- mice were infected intranasally with 1×10^9^ CFU of P. aeruginosa strain 8821 or an equivalent volume of saline (NT). BALF and Lung were collected 24 hpi. Macrophage (A) and platelet (B) recruitment to the BALF was assessed by Flow Cytometry. Neutrophil recruitment to the lung (C) were assessed by MPO activity. The upper lobe of the left lung was collected for H&E staining (D, original magnification × 200 for panels a-d) (n = 9 ± SEM). (TIF) [file pone.0205521.s005.TIF]

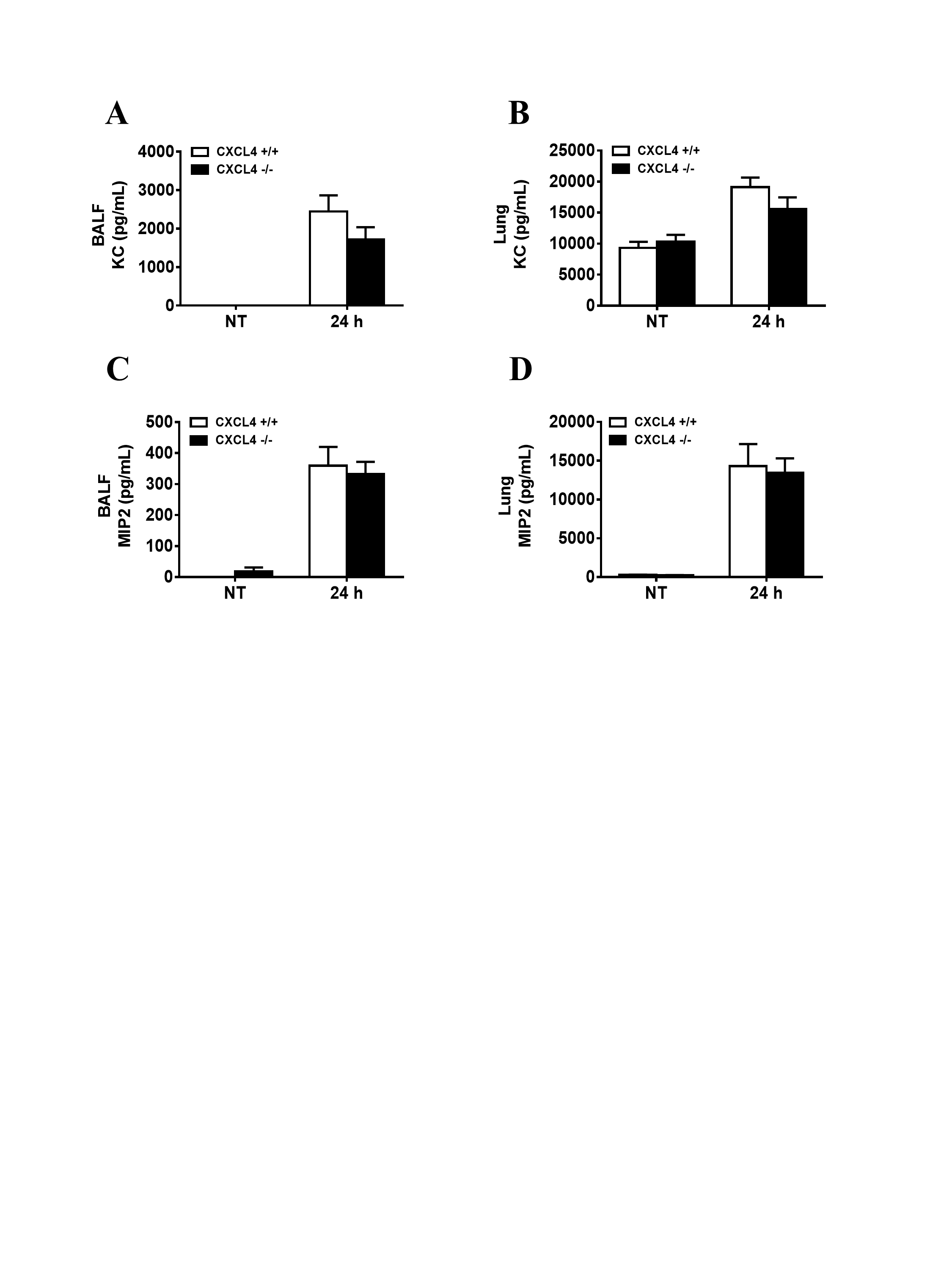

Supplement: S6 Fig — Wild-type and CXCL4-/- mice were infected intranasal with 1×10^9^ CFU of P. aeruginosa strain 8821 or an equivalent volume of saline (NT). Lung and BALF were collected 24 hpi. Supernatants of BALF and lung homogenate were analyzed for production of KC and MIP2 (n = 8 ± SEM). (TIF) [file pone.0205521.s006.TIF]
